# Prior multisensory learning can facilitate auditory-only voice-identity and speech recognition in noise

**DOI:** 10.1177/17470218241278649

**Published:** 2024-09-20

**Authors:** Corrina Maguinness, Sonja Schall, Brian Mathias, Martin Schoemann, Katharina von Kriegstein

**Affiliations:** 1 Chair of Cognitive and Clinical Neuroscience, Faculty of Psychology, Technische Universität Dresden, Dresden, Germany; 2 Max Planck Institute for Human Cognitive and Brain Sciences, Leipzig, Germany; 3 School of Psychology, University of Aberdeen, Aberdeen, United Kingdom; 4 Chair of Psychological Methods and Cognitive Modelling, Faculty of Psychology, Technische Universität Dresden, Dresden, Germany

**Keywords:** Speech in noise, voice identity, person recognition, audio–visual, multisensory learning

## Abstract

Seeing the visual articulatory movements of a speaker, while hearing their voice, helps with understanding what is said. This multisensory enhancement is particularly evident in noisy listening conditions. Multisensory enhancement also occurs even in auditory-only conditions: auditory-only speech and voice-identity recognition are superior for speakers previously learned with their face, compared to control learning; an effect termed the “face-benefit.” Whether the face-benefit can assist in maintaining robust perception in increasingly noisy listening conditions, similar to concurrent multisensory input, is unknown. Here, in two behavioural experiments, we examined this hypothesis. In each experiment, participants learned a series of speakers’ voices together with their dynamic face or control image. Following learning, participants listened to auditory-only sentences spoken by the same speakers and recognised the content of the sentences (speech recognition, Experiment 1) or the voice-identity of the speaker (Experiment 2) in increasing levels of auditory noise. For speech recognition, we observed that 14 of 30 participants (47%) showed a face-benefit. 19 of 25 participants (76%) showed a face-benefit for voice-identity recognition. For those participants who demonstrated a face-benefit, the face-benefit increased with auditory noise levels. Taken together, the results support an audio–visual model of auditory communication and suggest that the brain can develop a flexible system in which learned facial characteristics are used to deal with varying auditory uncertainty.

## Introduction

**Figure 1.**
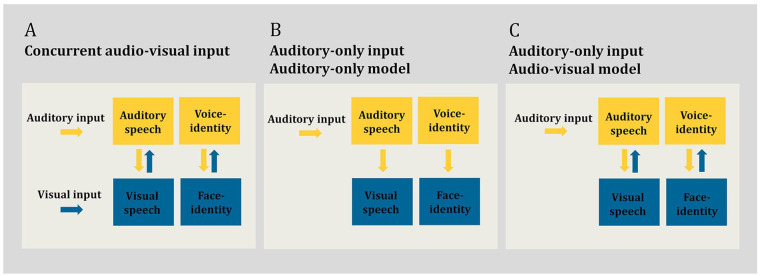
Schematic overview of auditory–visual processing in human communication. (a) Concurrent audio–visual input. In this listening condition, the listener can hear the speaker’s voice and see the speaker’s face concurrently. Processing of the auditory signal (auditory speech and voice identity) is supported by interactions (indicated via bidirectional arrows) between visual and auditory systems (Peelle & Sommers, 2015; Young et al., 2020). Here, concurrent visual cues help to predict and enhance the sensory processing of the auditory signal, this is particularly beneficial in noisy listening conditions (Ross et al., 2007; Sumby & Pollack, 1954). (b) Auditory-only input processed in an auditory-only model. In this listening condition, the listener can hear the speaker’s voice only, e.g., on the phone. The speaker’s face is not available. The speaker is, however, known to the listener audio–visually, such as a familiar person. The auditory system is engaged in the sensory processing of the auditory signal (auditory speech and voice identity) (Ellis et al., 1997; Hickok & Poeppel, 2007). Any engagement of the visual system, including for speakers known by face, is epiphenomenal to the processed auditory signal (indicated via unidirectional arrows) (Bunzeck et al., 2005). Under this model, learned visual mechanisms are not behaviourally relevant for auditory-only processing and, consequently, would not benefit sensory processing in noisy listening conditions. (c) Auditory-only input processed in an audio–visual model. The listening condition is identical to Panel B, i.e., auditory-only input. That is, again only the speaker’s voice is available to the listener, such as on the phone. The speaker is, however, known to the listener audio–visually, such as a familiar person. Both the auditory and visual system are engaged in the sensory processing of the auditory signal (auditory speech and voice identity) (von Kriegstein, 2012; von Kriegstein et al., 2008). Interactions between the systems (indicated via bidirectional arrows) are behaviourally relevant: in a similar manner to concurrent visual input (see Panel A), learned visual mechanisms (for speakers known by face) assist in auditory processing by generating predictions and providing constraints about what is heard in the auditory signal (von Kriegstein et al., 2008). Such a process should be particularly beneficial in noisy listening conditions. In Panels A, B, and C, yellow colour denotes the auditory system, blue colour denotes the visual.

Although speech is primarily conveyed acoustically, there is a large body of evidence that speech recognition is improved when the listener can both hear and see the speaker (see Peelle & Sommers, 2015 for review). These multisensory improvements are particularly apparent when the auditory signal is degraded, for example, in the presence of acoustic noise (Erber, 1969; Grant & Seitz, 2000; Rosenblum et al., 1996; Ross et al., 2007; Sumby & Pollack, 1954). Parametric increase in the noise level in the auditory signal *increases* the benefit of concurrent visual, in addition to auditory, speech input. This is particularly the case in low auditory signal-to-noise ratios (SNRs) (Erber, 1969; Ross et al., 2007; Sumby & Pollack, 1954), with a maximum multisensory benefit observed at specific SNRs (e.g., −12 dB see Liu et al., 2013; Ross et al., 2007). This multisensory benefit is thought to occur because the speaker’s visible dynamic articulatory movements are correlated with the production of the auditory speech signal (Campbell, 2008; Chandrasekaran et al., 2009; Rosenblum et al., 1996; Scholes et al., 2020). Visual articulatory movements can assist in generating predictions about what is to be heard in the auditory speech signal (Arnal et al., 2009; Bourguignon et al., 2020; Chandrasekaran & Ghazanfar, 2013; Davis et al., 2008; Jääskeläinen et al., 2004; Maguinness et al., 2011; Peelle & Sommers, 2015; Van Wassenhove et al., 2005), thereby enhancing speech recognition in challenging listening conditions (Figure 1a).

Perhaps surprisingly, and contrary to conventional views of auditory processing (auditory-only model) outlined in Figure 1b (Ellis et al., 1997; Hickok & Poeppel, 2007; see von Kriegstein, 2012 for review), there is evidence that the auditory sensory system continues to exploit visual mechanisms even under *auditory-only* listening conditions (Figure 1c). Listeners are more accurate at recognising the auditory-only speech utterances of speakers whose voices have been *previously* learned while paired with their corresponding face, in comparison to an audio–visual control learning condition (Riedel et al., 2015; Schelinski et al., 2014; von Kriegstein et al., 2008). In parallel, another aspect of auditory communication—voice-identity recognition (also referred to as speaker recognition)—is enhanced following audio–visual voice–face learning. Listeners are superior at recognising the identity of voices that have been previously learned together with the speaker’s face, in comparison to several auditory-only and audio–visual control learning conditions (Maguinness & von Kriegstein, 2021; Schall et al., 2013; Schelinski et al., 2014; Sheffert & Olson, 2004; von Kriegstein et al., 2008; von Kriegstein & Giraud, 2006; Zadoorian & Rosenblum, 2023; Zäske et al., 2015). The behavioural enhancement seen for both speech and voice-identity recognition in auditory-only listening conditions has been termed the “face-benefit” (von Kriegstein et al., 2008). As such, the face-benefit is a difference score and reflects the positive impact of *prior* audio–visual voice–face learning on subsequent auditory-only processing. For both auditory-only speech and voice-identity recognition, the face-benefit emerges rapidly and it is evident in auditory-only processing when the participant has previously had circa 2 min of audio–visual exposure to the speaker’s identity (Schall et al., 2013; Schelinski et al., 2014; von Kriegstein et al., 2008). The face-benefit is observed in the majority of neurotypical participants, that is, 76% for auditory voice-identity recognition and 58% for auditory speech recognition (von Kriegstein et al., 2008).

Face-benefits in auditory speech and voice-identity recognition have been explained under an audio–visual model of human auditory communication, shown in Figure 1c (see von Kriegstein, 2012 for review; von Kriegstein et al., 2005, 2008; von Kriegstein & Giraud, 2006). In this view, face-benefits are driven by a generative model (Kiebel, 2009; Mathias & von Kriegstein, 2023) of the learned speaker. Specifically, a face-benefit in auditory-only processing likely arises because, during audio–visual exposure, sensory processing becomes rapidly tuned to the spatiotemporal relationship between the auditory speech signal and the dynamic (e.g., lip movements) and static (i.e., structural form) face features, in a speaker-specific manner. This presumably does not occur when voices are paired with arbitrary control visual stimuli during learning (von Kriegstein et al., 2008; von Kriegstein & Giraud, 2006). Subsequently, even in the absence of concurrent visual facial input, the sensory system can exploit this learned non-arbitrary sensory coupling. For auditory-only speech processing, the system can simulate the missing *dynamic* visual trajectory of the auditory signal. For voice-identity recognition, the static features of the face are simulated (von Kriegstein et al., 2008).

In accordance with the audio–visual model of auditory communication (Figure 1c), functional magnetic resonance imaging (MRI) studies demonstrate that the left posterior superior temporal sulcus motion-sensitive face area (pSTS-mFA) is recruited during the recognition of auditory-only speech utterances of face-learned speakers (von Kriegstein et al., 2008). The pSTS-mFA is engaged in processing dynamic visual speech movements and this region is thought to forward information to auditory speech-processing regions during concurrent audio–visual input, that is, when the face and voice of the speaker are available (Arnal et al., 2009; Blank & von Kriegstein, 2013; Van Wassenhove et al., 2005). The observation that such visual cortices are also recruited during auditory-only speech processing, for speakers known by face, highlights that the brain uses similar visual mechanisms to support speech processing regardless of whether concurrent visual input is available. Responses in the left pSTS-mFA under auditory-only listening conditions likely reflect behaviourally relevant simulations of the dynamic visual trajectory of the speech signal. This is supported by the findings that inhibitory stimulation of the left pSTS-mFA *reduces* the behavioural face-benefit for auditory-only speech recognition (Riedel et al., 2015). Such findings are contrary to the auditory-only model (Figure 1b), which assumes that engagement of the visual system during auditory-only processing is not relevant for performing auditory tasks. Conversely, responses in the fusiform face area (FFA)—a region implicated in processing static structural face cues which support face-identity processing (Axelrod & Yovel, 2015; Eger et al., 2004; Ewbank & Andrews, 2008; Grill-Spector et al., 2004; Kanwisher et al., 1997; Kanwisher & Yovel, 2006; Liu et al., 2010; Rotshtein et al., 2005; Weibert & Andrews, 2015)—are apparent during the recognition of speakers known by face (Schall et al., 2013; von Kriegstein et al., 2008; von Kriegstein & Giraud, 2006). These FFA responses are behaviourally relevant: they positively correlate with the magnitude of the face-benefit for voice-identity recognition (Maguinness & von Kriegstein, 2021; von Kriegstein et al., 2008). Cross-modal interactions between the visual face-processing regions (left pSTS-mFA, FFA) and the respective auditory regions (left anterior superior temporal gyrus/sulcus, temporal voice areas in the right superior temporal gyrus/sulcus, are also readily observed during auditory-only speech and voice-identity processing (Schall & von Kriegstein, 2014; von Kriegstein & Giraud, 2006; von Kriegstein et al., 2006).

Although the visual mechanisms that underpin the face-benefit for speech and voice-identity recognition are thought to be largely distinct (i.e., dynamic facial cues for speech and static for identity), the governing principles for such cross-modal interactions are assumed to be the same: Visual mechanisms assist subsequent auditory-only recognition by generating predictions about, and thus placing constraints on the processing of, the incoming auditory signal (Blank et al., 2015; von Kriegstein et al., 2008). Such a process would be particularly beneficial for optimising recognition when the auditory signal is weak or degraded, with predictions assisting recognition by “filling in” missing sensory information. Notably, there is evidence that the face-benefit for voice-identity recognition persists in noisy auditory-only listening conditions. Maguinness and von Kriegstein (2021) demonstrated that, in an MRI environment, listeners show a face-benefit (16 of 21 participants tested) in recognising the identity of voices at both +4 and −4 dB SNRs. Moreover, for individuals who displayed a face-benefit, responses in the right FFA correlated positively with the behavioural face-benefit score at a low noise level (i.e., SNR + 4 dB). This observation of persistent behaviourally relevant FFA responses in noise fits well with magnetoencephalography recordings of early FFA responses, ~110 ms after auditory onset, for speakers learned by face (Schall et al., 2013). Notably, these responses are at a time point when voice-identity recognition has yet to occur in the human brain (Schweinberger, 2001), suggesting that visual mechanisms play a role in the sensory processing of the voice (Maguinness & von Kriegstein, 2021; Schall et al., 2013). However, it remains unknown whether a face-benefit for speech is also evident in noisy listening conditions, akin to the advantages observed with concurrent audio–visual input (Ross et al., 2007; Sumby & Pollack, 1954). Moreover, it is unclear if face-benefits for speech and voice-identity recognition scale with increasing noise in the auditory signal (i.e., with parametric modulation of the SNR in the auditory speech signal). The aim of the studies presented here was to test these hypotheses: that voice–face learning benefits speech and voice-identity recognition in noise and that noise levels can predict the magnitude of the face-benefit. Such observations would provide critical support for an audio–visual model of human auditory communication by demonstrating that cross-modal visual mechanisms play a similar role as concurrent visual input in supporting auditory-only processing (Figure 1c).

To test our hypotheses, we conducted two behavioural experiments in which we examined the effects of multisensory audio–visual exposure on subsequent auditory-only recognition in noisy listening conditions. We adopted a parametric design similar to previous reports on the effect of concurrent auditory and visual speech inputs (Erber, 1969; Liu et al., 2013; Ross et al., 2007; e.g., Sumby & Pollack, 1954). We tested whether the face-benefit increased with decreasing SNRs of the auditory signal. Specifically, we examined the face-benefit for auditory-only speech (Experiment 1) and voice-identity (Experiment 2) recognition performance across four levels of increasing auditory noise (SNRs: +4, 0, −4, and −8 dB). In both experiments, the speakers were learned prior to auditory-only testing. Crucially, half of the speakers were learned by seeing and listening to the videos of the speaker talking (voice–face learning); the other half were learned by listening to the speaker while viewing a visual control image depicting the speaker’s occupation (voice–occupation learning). First, replicating previous findings (Schall et al., 2013; Schelinski et al., 2014; Sheffert & Olson, 2004; von Kriegstein et al., 2008), we expected to observe a general benefit for audio–visual learning on auditory-only speech and voice-identity recognition, that is, the face-benefit would be present regardless of the noise level tested. Moreover, our second and central hypothesis was that the face-benefit would increase with decreasing SNRs in the auditory signal. Such a finding would indicate that learned visual mechanisms can help to systematically resolve incoming noisy auditory input. We expected this effect to be evident for both speech (Experiment 1) and voice-identity (Experiment 2) recognition.

## Experiment 1

The studies reported here were approved by the Ethics Committee of the Medical Faculty at the University of Leipzig, Germany (277-09-141209) and were conducted in accordance with the World Medical Association Declaration of Helsinki. All participants gave written informed consent prior to participation.

### Materials and methods

#### Participants

The required sample size to detect a potential difference between the voice–face and voice–occupation learning conditions (i.e., difference between two dependent means) for auditory-only speech recognition in Experiment 1 was calculated using GPower 3.1 (Faul et al., 2007, 2009) with the following parameters: effect size Cohen’s *d_z_* = 0.66 (Schelinski et al., 2014), α = .05, power (1−α) = 0.95. Note that the difference score used for Cohen’s *d_z_* calculation was based on the face-benefit score in auditory-only speech recognition (i.e., performance for voice–face-learned speakers *minus* voice–occupation-learned speakers) at SNR + 3 dB (the only SNR tested) for the typically developed sample in the work by Schelinski et al. (2014). The required sample size for Experiment 1 was calculated as 27 participants.

Thirty right-handed (Oldfield, 1971) participants (13 females, *M*_age_ = 24.8 years, *SD* = 2.6 years, range: 20–30 years) completed Experiment 1 and were included in the analyses. Participants were recruited from the participant database of the Max Planck Institute for Cognitive and Brain Sciences (Leipzig, Germany) and the participant management system of the Technische Universität Dresden (Dresden, Germany). All were native German speakers and all reported normal, or corrected to normal, vision and normal hearing. Seven additional participants were recruited but did not complete the experiment because they failed to reach the predefined threshold of ⩾ 80% correct (task: match the voice to a name and face/occupation image) during the audio–visual training stage (see “Audio–visual training” in the “Procedure” section below for full details) and therefore did not proceed to the auditory-only test phase of the experiment. As such, a larger number of participants were recruited than the required sample size (for Experiment 1 and 2), as it was anticipated that not all would pass the audio–visual training stage.

#### Stimuli and apparatus

##### Audio–visual training stimuli

The stimuli consisted of video recordings of six male native German speakers (22–30 years old). The videos were recorded using a high-definition camera (HDLegria HF S10, Canon, Japan) and an external microphone. Video stimuli were edited in Final Cut Pro software (Apple Inc., California, USA) to include a circular-centred mask, which excluded the background while revealing the face of the speaker. Videos were cropped to 727 × 545 pixels. In addition, for each speaker, a single frame was extracted from the video sequence, this image was used for the evaluation stage (see Audio–visual training, Training procedure section). The image contained the speaker in a neutral non-speech pose, that is, with the mouth closed. Three symbols representing an occupation were taken from Clip Art (http://office.microsoft.com/en-us/). The stimuli for the audio–visual *voice–face* training phase consisted of five video and three auditory recordings (per speaker) of questions which were five to six words in length and each approximately 2.5 s (s) in duration (e.g., “Trägt der Junge einen Koffer?,” English: “Is the boy carrying a suitcase?”). The stimuli for the audio–visual *voice–occupation* training phase were identical to that used for the *voice–face* training with the exception that the dynamic video image was replaced with one of the Clip Art pictures.

##### Auditory-only speech recognition stimuli

The stimuli were auditory recordings of sentences spoken by the same six male speakers (22–30 years of age) from the audio–visual training. The auditory stimuli were recorded in a soundproof room using high-quality recording equipment (Microphone: TLM 50, Neumann, Berlin; Mic-Preamp: Mic-Amp F35, Lake People, Germany; Soundcard: Power Mac G5 Dual 1.8 GHz, Apple Inc., CA, USA; Software: Sound Studio 3, Felt Tip, Inc., NY, USA) (44.1 kHz; 16 bit, mono). The stimuli were adjusted for overall mean amplitude using MATLAB version 7.0 (MathWorks, MA, USA). The sentences used in the auditory-only speech recognition test phase were 30 statement sentences that were five to six words in length (e.g., “Der Mann geht am Stock,” English: “The man walks with a cane”), each with a mean duration of approximately 2–2.5 s. Each speaker uttered each sentence. These auditory-only test stimuli (65 dB sound pressure level) were mixed with pink noise (created in MATLAB version 7.0 by filtering Gaussian white noise) of varying intensities to produce SNRs of +4, 0, −4, and −8 dB. An SNR of +4 dB indicates that the signal volume is 4 dB higher than the noise volume, while an SNR of −4 dB indicates that the noise volume is 4 dB higher than the signal. These SNRs and intervals (i.e., −4 dB steps) are comparable to previous audio–visual speech-in-noise studies (0, −4, and −8 dB see Liu et al., 2013; Ross et al., 2007). Based on pilot testing in a different participant sample (*N* = 6), we did not include lower SNRs as they produced close to chance speech recognition performance (SNR −10 dB, *M* = 57% correct utterance recognition accuracy). The pilot testing used a similar task design as the main experiment, but participants were exposed to only one noise level during auditory-only testing. The SNR of +4 dB was included to replicate previous studies on the face-benefit conducted outside the MRI environment, which used an SNR of +3 dB (Riedel et al., 2015; Schall et al., 2013; Schelinski et al., 2014). The pink noise was ramped and introduced with a linear 50-ms fade-in and fade-out. The experiment was programmed in and run using the Presentation software (Neurobehavioral Systems, Inc., California, USA). It was presented on a 19-in Samsung SyncMaster CRT monitor (Samsung Electronics Co., Ltd., Suwon, South Korea). All auditory stimuli were presented binaurally via Sennheiser HD 280 pro headphones (Sennheiser Electronic GmbH & Co. KG, Hannover, Germany).

#### Design

The experiment was based on a within-subject factorial design with learning (voice–face learned, voice–occupation learned) and noise level (SNR +4, 0, −4, −8 dB) as repeated factors. Learning was implemented using an established audio–visual training protocol which has repeatedly demonstrated the face-benefit in previous studies (Schall et al., 2013; Schelinski et al., 2014; von Kriegstein et al., 2008). Note as per the current study, these previous studies have used only male speakers. As we were specifically interested in the effect of noise level on the face-benefit, we kept the learning design as comparable as possible to the previous studies. The dependent variable was response accuracy for recognising auditory-only speech utterances. To investigate a potential speed-accuracy trade-off, we also analysed response times (see the online Supplementary Material for response time analyses and results).

#### Procedure

##### Audio–visual training

**Figure 2.**
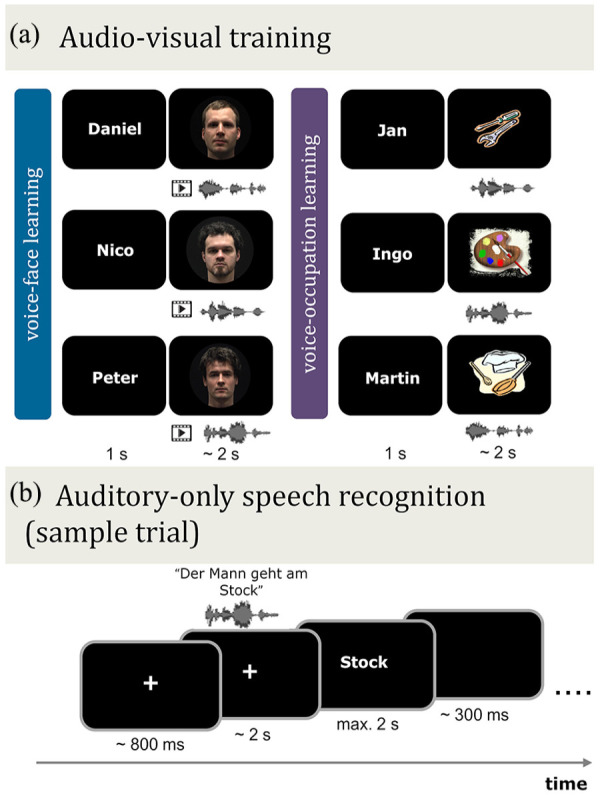
Schematic illustration of the audio–visual training phase and auditory-only speech recognition test phase in Experiment 1. (a) Audio–visual training. Prior to auditory-only testing, participants learned the voice and name of six speakers in conjunction with their corresponding face, i.e., video (voice–face learning) or with an occupation image (voice–occupation learning). (b) Auditory-only speech recognition. Following audio–visual training, participants listened to auditory-only sentences spoken by the same learned six speakers in different levels of auditory noise (SNR +4, 0, −4, −8 dB). The sentences were presented in blocks (15 trials per block), which were blocked by learning type (voice–face learned speakers or voice–occupation learned speakers) and noise level. In each trial, participants viewed a fixation cross and then listened to a speaker utter a five- or six-word sentence. This was followed immediately by the presentation of a word on screen. The participant’s task was to decide if the presented word was contained within the previously heard sentence or not. Note that the face identities shown in (a) are for illustration purposes, and some differ from those used in the audio–visual training phase. These images are not displayed due to consent restrictions.

*Training conditions*: All participants were familiarised with the same six speakers. Participants learned three of the speakers together with their corresponding face-identity (voice–face learning) and three with a visual symbol depicting their occupation (voice–occupation learning). In both conditions, participants also learned the speaker’s name (Figure 2a). The three speakers assigned to the voice–face learning or voice–occupation learning conditions were counterbalanced across participants. That is, Participant 1 learned Speakers 1, 2, and 3 in the voice–face learning condition, while Participant 2 learned Speakers 1, 2, and 3 in the voice–occupation learning condition (vice versa for Speakers 4, 5, and 6) and so on. Note these speaker sets always contained the same speaker identities, namely, Speakers 1, 2, and 3 or Speakers 4, 5, and 6. These learning conditions were used because they have been shown to reveal a replicable face-benefit across studies (Schelinski et al., 2014; von Kriegstein et al., 2008).

*Training procedure*: Familiarisation with the speakers was achieved through a series of training rounds. Each round consisted of a learning stage, followed by an evaluation stage. In the learning stage, participants were exposed to two blocks of audio–visual trials containing the five- to six-word question clips of speakers paired with either their corresponding face-identity (voice–face learning) or with an occupation symbol (voice–occupation learning). The speaker sets, that is, voice–face or voice–occupation, were always presented in separate blocks and the starting order of voice–face and voice–occupation learning was counterbalanced across participants. Each block consisted of three speakers uttering five sentences of five to six words. The name of the speaker appeared on screen before the audio–visual speaker was presented. Participants were instructed to memorise the names, voices, and faces/occupations well, but not to respond. Each speaker was heard saying each sentence once, amounting to 15 trials per block. Trials within a block were presented in a randomised order and were interleaved with an inter-trial interval of 1 s.

In the evaluation stage, learning was assessed. The evaluation stage occurred after each audio–visual speaker block. The criterion for reliably completing the evaluation stage was preset at ⩾80% correct, identical to the threshold used in the work by Schall et al. (2013), Schelinski et al. (2014), and von Kriegstein et al. (2008). During evaluation, participants completed 18 trials in which they matched a visually presented name or face/occupation image to the voice of a speaker (learned in the preceding block). In each trial participants listened to one of the three sentences (five- to six-word questions), which was immediately followed by either a name or a face/occupation image. Each speaker was heard uttering each of the three sentences two times, amounting to six trials per speaker. Sentences used in the evaluation stage were not presented during audio–visual learning. On half of the trials, the voice matched the name or image, while the remaining trials contained mismatched, i.e., distractor, names/images. The distractor names and images were always taken from the same speaker set (i.e., voice–face or voice–occupation). Participants indicated via button press whether the voice and the name or face/occupation belonged to the same speaker (“yes” or “no”). The name or image remained on screen until a response was made. Participants received written on-screen feedback (correct/incorrect) for 500 ms after a response was made. This was followed by a presentation of the correct name and voice–face or voice–occupation pairing. All participants completed at least two rounds of training. If after two rounds participants could reliably match the correct combinations of voice, name, and face/occupation (criterion: ⩾ 80% correct), they began the auditory-only speech recognition test stage (see next section). Thus, training consisted of 60 learning trials (15 trials per block × 4 blocks) and evaluation of 72 trials (18 trials per block × 4 blocks) for participants who needed two rounds to reach the criterion. Those with < 80% correct completed another training round, if they failed to reach the criterion on completing the third training round, they were excluded from the experiment and did not complete the recognition test phase.

##### Auditory-only speech recognition

The auditory-only speech recognition test served to evaluate the face-benefit. In the test, participants listened to the sentences uttered by the same six speakers who had been learned during the audio–visual training phase. Each trial started with the presentation of a fixation cross in the centre of the screen. Then (random jitter 500–1,000 ms) the auditory stimulus of a speaker uttering a five- to six-word statement sentence was presented. The sentence was followed by a visually presented word. Participants indicated via button press, “yes” or “no,” whether the word was present in the preceding sentence. Each word was displayed until a response was made, up to a maximum of 2 s (Figure 2b). After a response was made (or after 2 s if there was no response), the word disappeared, and the screen stayed blank for 300 ms until the next trial began. In half of the trials, the displayed word matched a word that had occurred within the preceding sentence. In the remaining trials, the word was an incorrect match. Words for the incorrect trials were chosen for either their semantic or phonological similarity to those in the sentences. For example, for the sentence, “Der Mann geht am Stock,” English: “The man walks with a cane” the presented correct words could be “Mann,” “geht,” or “Stock,” English: “man,” “walks,” “cane” and for the incorrect words “Kranke,” “steht,” or “Dock,” English: “sick person,” “stands,” or “dock.” The sentences were presented in four different levels of auditory noise (SNR +4, 0, −4, −8 dB). There were 720 trials in total: each of the six speakers uttered each of the 30 sentences once in four levels of auditory noise (6 × 30 × 4). Trials were presented in 48 separate blocks, each containing 15 trials. Each block was grouped by speaker type (i.e., voice–face or voice–occupation), and speaker identities within a block were presented in a randomised order. Sentences in each block were drawn randomly from the 30-sentence set. Each block contained one level of auditory noise (SNR +4, 0, −4, or −8 dB) and the block order of the noise levels was presented in a randomised order. After 12 blocks, the participants were given a rest period of 60 s.

A brief practice period proceeded the auditory-only speech recognition test. To refresh participants’ memory of the speakers, participants were presented with one audio–visual presentation of each speaker and their corresponding name, in the absence of auditory noise. This was followed by six practice trials to familiarise the participant with the task and introduce them to the auditory noise. In the practice trials, the participant was asked to recognise the speech utterances of the six learned speakers (procedure as in the main task) in the highest noise level (i.e., SNR −8 dB). Participants could repeat these six practice trials until they were comfortable with the testing procedure.

### Data analysis

The data from the auditory-only speech recognition trials were analysed. Trials in which the participant failed to respond (i.e., missed trials) were excluded from analyses as it cannot be ensured that the participant was attending to the presented auditory stimulus on these trials (a total of 0.27% of all trials, i.e., 59 of 21,600 trials across participants were excluded). Each participant’s overall trial count was then adjusted to include only trials for which a response was made. Accuracy (i.e., % correct) was calculated for each participant as the number of correctly identified words divided by the adjusted trial count × 100 for each learning condition (voice–face learned and voice–occupation learned), for each noise level (SNR +4, 0, −4, −8 dB). Response times (in ms) were also calculated for correct response trials, for each participant, for each learning condition (voice–face learned and voice–occupation learned) and noise level (see the online Supplementary Material for auditory-only speech recognition response time analyses and results).

To test our two hypotheses, we conducted linear mixed-effects (LME) regression analyses. The dependent variable was % correct for auditory-only speech recognition. The fixed effects were “learning” (voice–face learned, voice–occupation learned) and “noise level” (SNR +4, 0, −4, −8 dB). Learning was coded as a categorical fixed effect with two levels, that is, ANOVA-style coding (contrast weights −0.5, 0.5, with voice–occupation as the reference level) (Brehm & Alday, 2022). Based on prior research that the benefit of concurrent visual input on speech processing is positively related to increasing noise in the auditory signal and peaks at −12 dB SNR (Ross et al., 2007), noise level was treated as continuous fixed effect (i.e., 4, 0, −4, −8 dB). The random-effects structure was selected using backward model selection wherein random-effects terms that accounted for the least variance (zero or close to) were removed one by one until the model converged, that is, the fit was no longer singular.

We ran two LME regression models. The first model included all participants to assess our first hypothesis, that there is a general benefit for audio–visual voice–face, in comparison to voice–occupation, learning on auditory-only speech recognition across the group. This model also assessed whether the effect of learning may scale with increasing auditory noise in the general population sample. Based on prior studies (Maguinness & von Kriegstein, 2021; von Kriegstein et al., 2008), however, we expected that only a sub-sample of participants would show a benefit of audio–visual voice–face learning. Therefore, the second model included only the group of participants who showed an overall benefit of voice–face, in comparison to voice–occupation, learning. For this, we calculated face-benefit scores for each participant:

Face-benefit score = (% correct for voice–face-learned speakers) *minus* (% correct for voice–occupation-learned speakers), for each noise level.

Participants who had an overall positive mean face-benefit score (i.e., greater than zero across the four noise levels) were included in the second analysis. This analysis, restricted to those who benefitted from voice–face learning, that is, the face-benefit participant group, served to answer our second and central hypothesis on whether there was a *change* (i.e., increase) in the face-benefit with decreasing SNRs in the auditory signal.

For Experiments 1 and 2, all LME analyses were conducted using the “lme4” package (Bates et al., 2015) in R software (https://www.r-project.org/). The model was fit using maximum likelihood, and significance testing was performed using Satterthwaite’s method implemented in the “lmerTest” package, with an alpha level of α = .05 (Kuznetsova et al., 2017). Tukey-corrected post hoc pairwise comparisons were conducted using the “emmeans” package in *R* (Lenth, 2023).

### Results

#### Accuracy: auditory-only speech recognition

##### Model 1: all participants

An LME model on accuracy scores for all participants (*N* = 30) revealed an effect of noise level (*B* = 0.27, *SE* = 0.03, *t* = 8.12, *p* < .001, 95% CI = [0.20, 0.33]), which was driven by poorer performance at SNR −8 dB (*M*_% correct_ = 94.83, *SD* = 4.18) compared to all other noise levels (all *p*s < .001). Thus, noise level manipulation significantly impacted auditory-only speech recognition performance. No effect of learning (*p* = .43), or interaction between the learning and noise level variables (*p* = .42) was observed.

The marginal *R*^2^ value of .18 indicated that the fixed effects of learning condition and noise level accounted for 18% of the variance, while the conditional *R*^2^ value of .37 revealed that variability attributed to the model’s random-effects structure, which included both a random intercept of participant and a participant-by-learning condition slope, was nearly equivalent to the variance explained by the fixed effects. These results point towards variability among participants in the effect of learning on auditory-only speech recognition, suggesting that individual differences in learning effects be taken into account. This observation aligns with prior research showing that learning effects vary across individuals (e.g., von Kriegstein et al., 2008). See Table 1 for the full model results and Table 2, left of table, for mean accuracy scores. Therefore, to test our central hypothesis, that noise levels can predict the magnitude of the face-benefit, we assessed auditory-only speech recognition accuracy outcomes for only those participants who showed a benefit following voice–face, in comparison to voice–occupation learning, that is, a face-benefit.

**Table 1.** Summary of the LME analysis testing the effect of learning condition and noise level on auditory-only speech recognition accuracy in Experiment 1 in all participants (*N* = 30).

Auditory-only speech recognition					
LME regression: Model 1 (all participants)					
Fixed effects	*B*	95% CI	*SE*	*t*	*p*
Intercept	97.86	[97.31, 98.40]	0.28	354.17	<.001
Learning	0.27	[−0.40, 0.95]	0.34	0.79	.43
Noise level	0.27	[0.20, 0.33]	0.03	8.12	<.001
Learning × noise level	0.05	[−0.08, 0.18]	0.07	0.80	.42
Random effects		Variance	*SD*		
Participant	Intercept	1.51	1.23		
	Learning	0.38	0.62		
*R* ^2^					
Marginal	.18				
Conditional	.37				

**Table 2.** Auditory-only speech recognition performance. Mean accuracy (% correct with standard deviations) for voice–face and voice–occupation learned speakers, for each of the four noise levels. Face-benefit scores, that is (% correct for voice–face-learned speakers) *minus* (% correct for voice–occupation-learned speakers), are also shown. Scores for all 30 participants are on the left of the table, scores for the 14 participants with a positive overall face-benefit score are displayed on the right.

	All participants (*N* = 30)				Face-benefit participants (*N* = 14)			
SNR	+4 dB	0 dB	−4 dB	−8 dB	+4 dB	0 dB	−4 dB	−8 dB
Voice–face	98.59 (1.54)	98.33 (1.72)	97.89 (1.32)	94.82 (4.22)	98.33 (1.78)	98.97 (1.34)	98.41 (1.05)	97.27 (2.38)
Voice–occupation	97.99 (1.72)	98.14 (1.38)	97.99 (1.84)	94.83 (4.21)	97.46 (1.65)	98.33 (0.95)	98.01 (1.81)	92.68 (4.79)
Face-benefit	0.60 (1.40)	0.19 (1.68)	−0.11 (1.76)	−0.01 (5.51)	0.87 (1.39)	0.64 (1.73)	0.40 (1.83)	4.59 (3.92)

##### Model 2: face-benefit participant group

**Figure 3.**
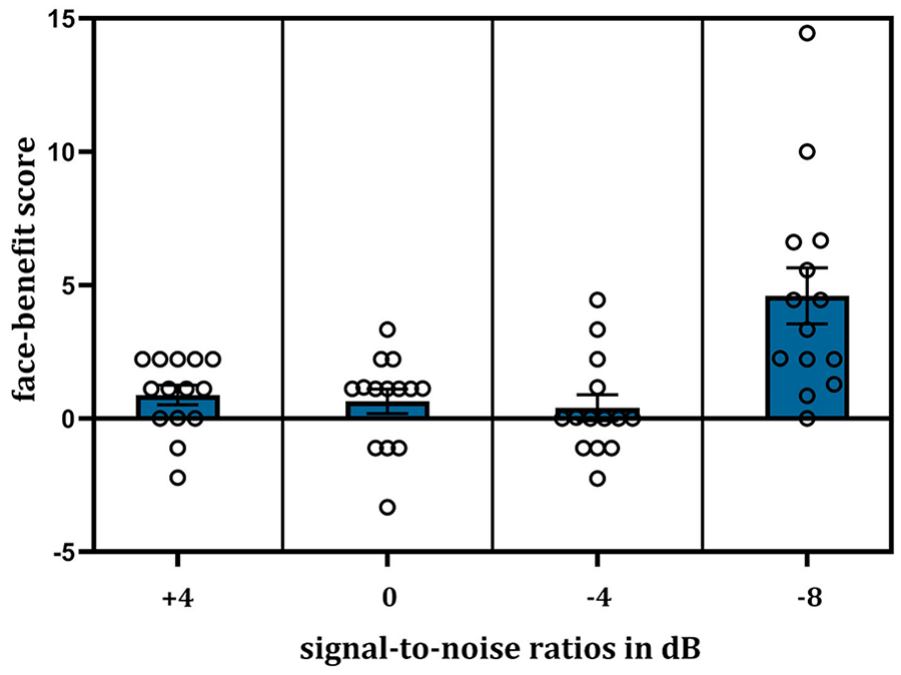
Plot showing the face-benefit score, that is (% correct voice–face-learned speakers) *minus* (% correct voice–occupation-learned speakers), for speech recognition (*N* = 14) with decreasing SNRs of the auditory signal. The face-benefit was most evident in the noisiest listening condition—SNR −8 dB. Error bars show ± 1 standard error of the mean. Individual participant scores are displayed as single points.

Fourteen of the 30 participants tested (47%) showed an overall benefit of voice–face learning on auditory-only speech recognition accuracy, that is, a positive face-benefit, averaged across noise levels. The model on accuracy scores for these face-benefit participants (*N* = 14) revealed an effect of learning (*B* = 1.08, *SE* = 0.47, *t* = 2.28, *p* = .025, 95% CI = [0.14, 2.02]), as expected in this sub-sample, auditory-only speech recognition was enhanced for speakers learned previously by face (*M*_% correct_ = 98.25, *SD* = 1.78), compared to speakers learned in the occupation (*M*_% correct_ = 96.62, *SD* = 3.52) condition. An effect of noise level was also evident (*B* = 0.23, *SE* = 0.05, *t* = 4.75, *p* < .001, 95% CI = [0.13, 0.33]), again with poorer performance in SNR −8 dB (*M*_% correct_ = 94.98, *SD* = 4.39) compared to all other noise levels (all *p*s < .001). As hypothesised, an interaction between the effects of learning and noise level was found (*B* = −0.27, *SE* = 0.10, *t* = −2.82, *p* = .006, 95% CI = [−0.46, −0.08]), see Table 3 for the full model results. As evidenced by the negative coefficient, the lower the SNR, the greater the benefit of voice–face, in contrast to voice–occupation, learning. However, on examination of the accuracy means (Table 2, right of the table), one can see that this linear effect of noise on learning appears to be driven by a large increase in the face-benefit at SNR −8 dB namely, in the noisiest listening condition. Post hoc analyses confirmed this: at SNR −8 dB, voice–face learned speakers (*M*_% correct_ = 97.27, *SD* = 2.38) were more accurately recognised than voice–occupation learned speakers (*M*_% correct_ = 92.68, *SD* = 4.79) i.e., a mean face-benefit of 4.59% (*SD* = 3.92) was evident. The face-benefit scores for each noise level are plotted in Figure 3.

**Table 3.** Summary of the LME analysis testing the effect of learning condition and noise level on auditory-only speech recognition accuracy in Experiment 1 in participants with a positive overall face-benefit (*N* = 14).

Auditory-only speech recognition					
LME regression: model 2 (face-benefit participants)					
Fixed effects	*B*	95% CI	*SE*	*t*	*p*
Intercept	97.89	[97.19, 98.59]	0.35	276.39	<.001
Learning	1.08	[0.14, 2.02]	0.47	2.28	.025
Noise level	0.23	[0.13, 0.33]	0.05	4.75	<.001
Learning × noise level	−0.27	[−0.46, −0.08]	0.10	−2.82	.006
Random effects		Variance	*SD*		
Participant	Intercept	0.97	0.98		
*R* ^2^					
Marginal	.25				
Conditional	.37				

### Discussion

The results revealed, in accordance with our hypothesis, that the benefit of voice–face, compared to voice–occupation learning, that is the face-benefit, increased with noise in the auditory signal. However, this was observed only for participants who exhibited an overall benefit for voice–face learning. At the whole group level, there was no significant impact of learning on auditory-only speech recognition. Notably, the number of participants who demonstrated a face-benefit was in line, although below, previous observations, namely 47% (current study) versus 58% (von Kriegstein et al., 2008). In the face-benefit participant group, this enhanced auditory-only speech recognition performance could not be explained by differences in response times when responding to voice–face-learned speakers relative to voice–occupation-learned speakers, that is, there was no speed–accuracy trade-off (see the online Supplementary Material for LME modelling results for response times). Although the LME regression model suggested that the magnitude of the face-benefit scaled with decreasing SNRs, post hoc analyses confirmed that this relationship was propelled by an increase in the face-benefit at the lowest SNR tested (SNR −8 dB) relative to all other SNRs.

The result that the face-benefit increases in noise, and that it is most apparent at a specific auditory SNR, is similar to the previous observations on speech processing in noise when concurrent visual input is available (Crosse et al., 2016; Liu et al., 2013; Ross et al., 2007). We will come back to this observation in the discussion of Experiment 2 and the general discussion. In Experiment 2, we addressed whether the auditory SNR can also predict the magnitude of the benefit of voice–face, in contrast to voice–occupation, learning in auditory-only *voice-identity* recognition.

## Experiment 2

### Materials and methods

#### Participants

The required sample size to detect a potential difference between the voice–face and voice–occupation learning conditions (i.e., difference between two dependent means) for Experiment 2 was again calculated using GPower 3.1 (Faul et al., 2007, 2009) with the following parameters: effect size Cohen’s *d_z_* = 0.79 (Schelinski et al., 2014), α = .05, power (1−α) = .95. The effect size Cohen’s *d_z_* was again for the typically developed sample in Schelinski et al. (2014). Note that the difference score used for the Cohen’s *d_z_* calculation was based on the face-benefit score in auditory-only voice-identity recognition (i.e., performance for voice–face-learned speakers *minus* voice–occupation-learned speakers) at SNR +3 dB (Schelinski et al., 2014). The required sample size was calculated as 19 participants. This is a lower required sample size than Experiment 1, as the higher effect size observed for the face-benefit in voice-identity recognition suggests that learning effects may be detected in smaller sample sizes.

Twenty-seven native German speakers, recruited from the Max Planck Institute for Cognitive and Brain Sciences participant database completed the second experiment. None of the participants had participated in Experiment 1. All were right-handed, as assessed by the Edinburgh Handedness Inventory (Oldfield, 1971) and reported normal hearing and normal, or corrected-to-normal, vision. Two participants who completed the experiment were excluded from analysis—one due to below chance performance in the auditory-only test phase of the experiment and another due to the technical failure which disrupted data saving during auditory-only testing. Seven additional participants were recruited; however, they failed to meet the predefined learning criterion during the audio–visual training phase of the experiment (⩾ 80% correct) and thus did not proceed to the auditory-only voice-identity recognition test phase. As such, the analyses included a total of 25 participants (17 female, *M*_age_ = 24.3 years, *SD* = 2.3 years, range 21–30 years).

#### Stimuli and apparatus

##### Audio–visual training stimuli

The stimuli for audio–visual training included video recordings of six male native German speakers. The stimuli were recorded and processed in the same manner as that reported in Experiment 1, with the exception that three of the speakers were from different male identities (age range of six speakers: 22–27 years old; stimulus set adapted from Schall et al., 2013, 2014). The stimuli for the audio–visual *voice–fac*e training comprised five videos and three auditory recordings (per speaker) of five- to six-word statement sentences with a mean length of 2–2.5 s (e.g., “Die Rose wächst im Garten,” English: “The rose grows in the garden”). Stimuli for the audio–visual *voice–occupation* training phase were identical to that of the voice–face training, with the exception that the visual video display was replaced with a static occupation symbol, shown for the same duration as the video sequence. As per Experiment 1, the three symbols representing an occupation were taken from Clip Art (http://office.microsoft.com/en-us/). See Figure 2a.

##### Auditory-only voice-identity recognition stimuli

The stimuli for the auditory-only voice-identity recognition test phase of the experiment consisted of 30 two-word sentences, recorded, and processed in an identical manner as reported in Experiment 1. The sentences always started with “Er” (English: “He”) and finished with a verb (e.g., “Er liest,” English: “He reads”). All sentences were approximately 1 s in length. Note that these shorter sentences have been found to be sufficient for voice-identity tasks and have been previously used to investigate the face-benefit for voice-identity in noise (Maguinness & von Kriegstein, 2021; Schall et al., 2013, 2015). As in Experiment 1, auditory-only test stimuli were mixed with pink noise of varying intensities to produce SNRs of +4, 0, −4, and −8 dB. The noise-mixing procedure and the apparatus used for stimulus presentation were the same as in Experiment 1.

#### Design

The design was the same as Experiment 1, with the exception that the dependent variable was response accuracy for auditory-only *voice-identity* recognition. As per Experiment 1, to investigate a potential speed–accuracy trade-off, response times were also analysed.

#### Procedure

##### Audio–visual training

The audio–visual training, including the training conditions and training procedure (i.e., the learning and evaluation stages), was the same as in Experiment 1.

##### Auditory-only voice-identity recognition

**Figure 4.**
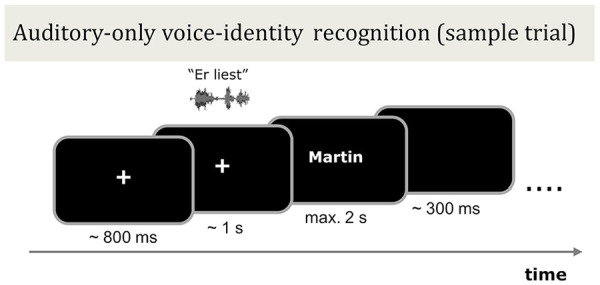
Schematic illustration of the auditory-only voice-identity recognition test phase in Experiment 2. Following audio–visual training with six male speakers (procedure identical to Figure 2a), participants listened to blocks of sentences spoken by the same speakers in different levels of auditory noise (SNR +4, 0, −4, −8 dB). Each block contained 15 trials. In each trial, participants viewed a fixation cross and heard the speaker utter a two-word sentence which was followed immediately by the presentation of a name on screen. The participant’s task was to decide if the presented name matched the identity of the speaker or not. The trials were blocked by learning type (voice–face learned speakers or voice–occupation learned speakers) and noise level.

The auditory-only voice-identity recognition test served to evaluate the face-benefit. In this test, listeners recognised the identity of the six previously learned speakers in four different levels of auditory noise. In each trial, a fixation cross was presented in the centre of the screen, this was then followed (random jitter 600–1,000 ms) by the auditory stimulus of a speaker uttering a two-word sentence. The name of one of the six learned speakers then immediately appeared on screen. Participants were instructed to indicate via button press, “yes” or “no,” whether the visually presented name matched the identity of the voice. The name was presented for a maximum of 2 s and participants were free to respond at any time during the name presentation. Once a response was made (or after 2 s for no response), the name disappeared from the screen and a blank screen was displayed for 300 ms. Participants were encouraged to respond on each trial. See Figure 4 for a sample trial structure.

The auditory-only voice-identity recognition test phase consisted of 720 trials in total. For half the trials, the visually presented name was correct, that is, matched the identity of the voice. For the other half of the trials, it was incorrectly matched. Incorrect names were always taken from the same speaker set. The trials were blocked and presented in the same manner as described in Experiment 1. After 12 blocks, participants were given a rest interval of 60 s. A brief practice period proceeded the auditory-only voice-identity recognition test. This practice period was identical to Experiment 1, with the exception that the task was to recognise the speaker identities.

### Data analysis

As in Experiment 1, trials in which a participant failed to make a response, that is, missed trials, were disregarded from analysis (a total of 2.63% of all trials across participants, i.e., 475 of 18,000 trials), and each participant’s overall trial count was adjusted accordingly. Accuracy (i.e., % correct) was calculated for each participant in an identical manner to Experiment 1: number of correct responses (auditory-only voice-identity recognition) divided by the adjusted trial count × 100 for each of the learning conditions (i.e., voice–face and voice–occupation learning), for each of the noise levels (SNR +4, 0, −4, −8 dB). All data (including response times, see the online Supplementary Material for response time analysis and results) were calculated and analysed using LME models in an identical manner as Experiment 1. In summary, the fixed effects were “learning” (voice–face learned, voice–occupation learned) and “noise level” (SNR +4, 0, −4, −8 dB), coded and treated as per Experiment 1. The random-effects structure was again selected using backward model selection. Random-effects terms that accounted for the least variance (zero or close to) were removed one by one until the model converged, that is, the fit was no longer singular. Again, we ran two LME regression models. The first model included all participants. The second model included only those participants who had an overall positive mean face-benefit score (i.e., greater than zero across the four noise levels). This second model, including the face-benefit participant group, served again to examine our second and central hypothesis on whether there was a *change* in the face-benefit as noise level increased. As per Experiment 1, the statistical significance threshold was set at α = .05.

### Results

#### Accuracy: auditory-only voice-identity recognition

##### Model 1: all participants

The LME model on accuracy scores across all participants (*N* = 25) revealed an effect of learning (*B* = 5.53, *SE* = 2.01, *t* = 2.75, *p* = .01, 95% CI = [1.56, 9.50]), such that speakers previously learned by face (*M*_% correct_ = 86.85, *SD* = 9.61) were more accurately recognised than those learned in the voice–occupation (*M*_% correct_ = 80.99, *SD* = 11.69) condition. An effect of noise level (*B* = 0.52, *SE* = 0.06, *t* = 8.13, *p* < .001, 95% CI = [0.39, 0.65]) was also found. Auditory-only voice-identity recognition performance was worse with increasing noise: performance at SNR −8 dB (*M*_% correct_ = 80.47, *SD* = 11.76) was worse than all other noise levels (all *ps* < .004). In addition, performance at SNR −4 dB (*M*_% correct_ = 83.34, *SD* = 10.42) was poorer than SNR +4 dB (*M*_% correct_ = 86.86, *SD* = 10.67) (*p* < .001). No interaction between the learning and noise level variables (*p* = .20) was observed. See Table 4, left of table, for mean accuracy scores.

**Table 4.** Auditory-only voice-identity recognition performance. Mean accuracy (% correct with standard deviations) for voice–face-learned and voice–occupation-learned speakers, for each of the four noise levels. Face-benefit scores, that is (% correct voice–face-learned speakers) *minus* (% correct voice–occupation-learned speakers), are also shown. On the left of the table, scores for all participants (*N* = 25) are displayed, on the right are scores for *N* = 19 face-benefit participants.

	All participants (*N* = 25)				Face-benefit participants (*N* = 19)			
SNR	+4 dB	0 dB	−4 dB	−8 dB	+4 dB	0 dB	−4 dB	−8 dB
Voice–face	89.72 (8.40)	87.10 (10.61)	86.52 (8.45)	84.06 (10.44)	91.74 (5.34)	90.08 (7.57)	88.27 (6.44)	86.88 (6.61)
Voice–occupation	83.99 (12.03)	82.93 (10.56)	80.15 (11.37)	76.88 (12.11)	83.16 (12.86)	81.44 (11.23)	78.31 (11.84)	75.38 (12.70)
Face-benefit	5.73 (10.96)	4.16 (12.02)	6.37 (11.00)	7.18 (11.54)	8.58 (10.57)	8.64 (9.40)	9.96 (9.77)	11.49 (8.92)

The marginal *R*^2^ value of .12 revealed that the fixed effects of learning condition and noise level accounted for 12% of the variance in the auditory-only voice-identity recognition task data, while the conditional *R*^2^ value of .87 indicated that the model’s random effects accounted for substantially more variance than fixed effects. Thus, the variability among participants in the effect of learning on auditory-only speech recognition was even larger in Experiment 2 than Experiment 1. The full model results are shown in Table 5. Therefore, as per Experiment 1, we conducted our second analysis on the group of participants who showed a benefit of voice–face, in contrast to voice–occupation, learning, i.e., those with a positive face-benefit (average across all noise levels). In this face-benefit participant group, we assessed whether the benefit of prior voice–face learning may scale with the degree of noise in the auditory signal.

**Table 5.** Summary of the LME analysis testing the effect of learning condition and noise level on auditory-only voice-identity recognition accuracy in Experiment 2 in all participants (*N* = 25).

Auditory-only voice-identity recognition					
LME regression: model 1 (all participants)					
Fixed effects	*B*	95% CI	*SE*	*t*	*p*
Intercept	84.96	[81.64, 88.28]	1.68	50.44	<.001
Learning	5.53	[1.56, 9.50]	2.01	2.75	.01
Noise level	0.52	[0.39, 0.65]	0.06	8.13	<.001
Learning × noise level	−0.16	[−0.42, 0.09]	0.13	−1.27	.20
Random effects		Variance	*SD*		
Participant	Intercept	68.46	8.27		
	Learning	91.38	9.56		
*R* ^2^					
Marginal	.12				
Conditional	.87				

##### Model 2: face-benefit participants

As expected, not all participants displayed a face-benefit. Rather, there was an overall positive face-benefit (i.e., greater than zero across the four noise levels) for 19 of the 25 participants tested. The LME model on auditory-only voice-identity accuracy scores in this face-benefit participant group (*N* = 19) revealed an effect of learning (*B* = 9.17, *SE* = 1.82, *t* = 5.03, *p* ⩽ .001, 95% CI = [5.56, 12.77]) as to be expected in this group. Speakers learned previously by face (*M*_% correct_ = 89.24, *SD* = 6.67) were more accurately recognised than speakers learned in the voice–occupation (*M*_% correct_ = 79.57, *SD* = 12.3) condition. See Table 4, right of table, for mean accuracy scores. An effect of noise level was also observed (*B* = 0.54, *SE* = 0.07, *t* = 7.20, *p* ⩽ .001, 95% CI = [0.39, 0.68]). This appeared to be driven by a systematic decrease in performance across multiple SNRs: performance at SNR −8 dB (*M*_% correct_ 81.13, *SD* = 11.56) was worse than all other noise levels (all *ps* < .001), except the adjacent SNR −4 dB (*p* = .12); SNR −4 dB (*M*_% correct_ = 83.29, *SD* = 10.67) performance trended towards poorer than that at SNR 0 dB (*p* = .055, *M*_% correct_ = 85.76, *SD* = 10.41) and was poorer than SNR + 4 dB (*M*_% correct_ = 87.45, *SD* = 10.64, *p* < .001). There was no interaction between learning and noise level (*p* = .09). Notably, however, voice-identity recognition was better for voice–face, than voice–occupation, learned speakers in each noise level tested, that is, a face-benefit was observed at each SNR (all *ps* < .01). See Table 4, right of table, for mean face-benefit scores per noise level. Again, the marginal and conditional *R*^2^ values indicated that variance explained by individual differences between participants in terms of the effect of learning on voice-identity recognition performance substantially exceeded variance explained by the model’s fixed effects, even in this group restricted to face-benefit participants. See Table 6 for full model results.

**Table 6.** Summary of the LME analysis testing the effect of learning condition and noise level on auditory-only voice-identity recognition accuracy in Experiment 2 for the face-benefit participant group (*N* = 19).

Auditory-only voice-identity recognition					
LME regression: model 2 (face-benefit participants)					
Fixed effects	Estimate	95% CI	*SE*	*t*	*p*
Intercept	85.47	[81.88, 89.08]	1.82	46.96	<.001
Learning	9.17	[5.56, 12.77]	1.82	5.03	<.001
Noise level	0.54	[0.39, 0.68]	0.07	7.20	<.001
Learning × noise level	−0.25	[−0.55, 0.04]	0.15	−1.69	.09
Random effects		Variance	*SD*		
Participant	Intercept	60.43	7.77		
	Learning	53.09	7.29		
*R* ^2^					
Marginal	.25				
Conditional	.86				

##### Model 3: face-benefit participants (normalised face-benefit scores)

To address this inter-participant variability and allow a comparison of the effect of voice–face, in contrast to voice–occupation, learning across participants, we proceeded to employ a normalisation procedure. For each face-benefit participant (*N* = 19), we adjusted their face-benefit score in each noise level relative to their overall performance, thereby providing a standardised metric of the benefit’s magnitude in each noise level that is directly comparable across individuals. Specifically, this *normalised face-benefit* score was calculated as: % correct voice–face minus % correct voice–occupation-learned speakers (i.e., face-benefit for each noise level), divided by the individual’s overall face-benefit score (i.e., the mean face-benefit across all noise levels). To illustrate, a normalised face-benefit of 0 indicates no face-benefit in that noise level; 0.5 indicates the face-benefit in that noise level is half the overall face-benefit; 1 indicates the face-benefit in that noise level is the same as the overall face-benefit; and 2 indicates that the face-benefit in that noise level is twofold the overall face-benefit (e.g., 8% in that given noise level compared to a 4% overall face-benefit), and so on.

**Figure 5.**
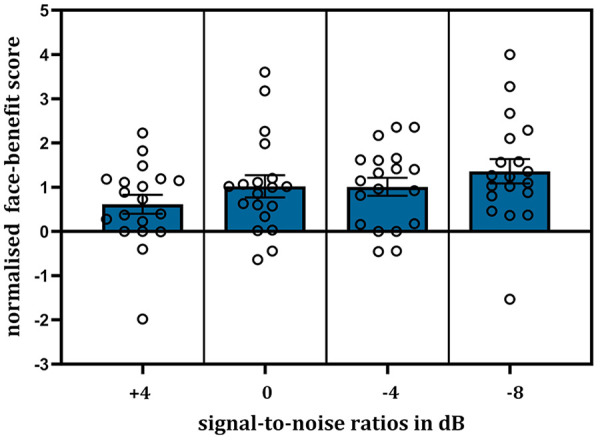
Plot showing the normalised face-benefit for voice-identity recognition across the increasing noise levels (*N* = 19). The increasing noise level significantly predicted the normalised face-benefit scores. Error bars show ± 1 standard error of the mean. Individual participant scores are displayed as single points.

We analysed the normalised face-benefit scores using LME modelling. The model included only the fixed effect of “noise level” (treated as a continuous variable, i.e., 0, 4, −4, −8 dB). Including the random effect of participant produced a singular fit, that is, the participant variable no longer explained any variance in outcomes due to the normalisation of face-benefit scores. The model results showed that an increase in noise level significantly predicted the normalised face-benefit scores (*B* = −0.06, *SE* = 0.03, *t* = −2.11, *p* = .038, 95% CI = [−0.11, −0.003]). In parallel, it explained a significant proportion of score variance, *R*^2^ = .06, *F*(1,74) = 4.45, *p* = .038. This systematic increase in the normalised face-benefit with increasing noise (i.e., decreasing SNRs) can be viewed in Figure 5.

### Discussion

The analysis of all participants revealed that while noise significantly impacted voice-identity recognition, speakers who were previously learned by face were, overall, more accurately recognised than those learned in the absence of a face (i.e., voice–occupation learning). When examining individual performance, 19 of the 25 tested participants displayed this face-benefit. The observation that 76% of participants exhibited a face-benefit aligns with prior observations in neurotypical participants regarding voice-identity processing (Maguinness & von Kriegstein, 2021; von Kriegstein et al., 2008). In these face-benefit participants, there was a face-benefit present in each noise level tested. Moreover, when participants’ face-benefit scores were normalised to provide a common scale to examine the magnitude of the face-benefit (in each noise level), there was evidence that the normalised face-benefit increased in parallel with the degree of noise in the auditory signal. This suggests a *systematic* benefit on auditory-only voice-identity processing for speakers learned by face. This benefit could not be explained by a speed–accuracy trade-off, that is, there was no evidence that voice–occupation-learned speakers were responded to more quickly (and hence less accurately). Rather face-benefit participants responded more accurately and in parallel more quickly to voices learned by face (see the online Supplementary Material for full LME analysis and results on the response times). These results differ qualitatively from the face-benefit for auditory-only speech recognition accuracy observed in Experiment 1 as, in Experiment 1, a face-benefit was found only at the lowest SNR of −8 dB. In the following general discussion, we explore in more detail potential explanations for the observed similarities and differences between Experiments 1 and 2.

## General discussion

The current study had two key findings. In Experiment 1, we found that, for individuals who displayed a face-benefit, the face-benefit for auditory-only speech recognition increased as the salience of the auditory signal decreased, with a benefit evident in the nosiest listening condition (SNR −8 dB). However, at the whole group level, there was no overall benefit of prior voice–face learning on auditory-only speech recognition. In Experiment 2, at the whole group level, an overall benefit of prior voice–face learning on auditory-only voice-identity recognition in noise was evident. For the individuals who showed a face-benefit, this benefit was evident in each level of auditory noise. Moreover, it systematically increased as auditory salience decreased when face-benefit scores in each noise level were expressed on a scale that allowed for direct comparison across individuals (i.e., normalised face-benefit). These findings are in line with our predictions and fit well with an audio–visual model of human auditory communication (von Kriegstein et al., 2008). In this view, learned facial characteristics are behaviourally relevant for supporting the sensory processing of the auditory signal, providing useful constraints which help to predict and resolve what is heard in the auditory domain (Figure 1c). Our findings support this view and demonstrate that the sensory system has developed a flexible mechanism to deal with auditory uncertainty even when visual cues are absent. Specifically, learned visual mechanisms may be recruited to enhance the recognition of auditory signals, boosting recognition when incoming sensory information lacks clarity. This observation challenges traditional auditory-only models of auditory processing (Figure 1b), which propose that recruitment of visual mechanisms is a by-product of successful auditory recognition. According to such models, one would not expect a face-benefit on auditory processing. Moreover, these models would not predict an increased reliance on visual mechanisms as auditory tasks become more difficult. In sum, our findings provide new evidence in favour of a holistic, multisensory approach to auditory recognition, particularly when navigating challenging listening environments.

### The face-benefit for speech increases in noise: parallels with speech processing involving concurrent audio–visual inputs

In terms of speech recognition, the face-benefit was most evident in the noisiest listening condition: SNR −8 dB. In this noise level, the face-benefit was approximately fourfold (~4.59%) what was observed in any other SNR tested (all < 1%). This large increase at a specific SNR parallels previous studies on the effects of concurrent visual input on speech recognition in noise (Liu et al., 2013; Ross et al., 2007). These studies, which have parametrically altered the noise level in the auditory signal, have shown that, while the benefit of visual input either increases linearly (Ma et al., 2009; Ross et al., 2007) or remains relatively consistent (T. Liu, 2007) with increasing noise at lower noise levels (up to SNR −8 dB), there is a “special zone” (Ross et al., 2007) where the multisensory benefit on speech recognition becomes maximal (Liu et al., 2013; Ma et al., 2009; Ross et al., 2007). This plateau in benefits of simultaneous auditory and visual speech presentation has been observed for what have been labelled as “intermediate” SNRs. For example, when the range of auditory SNRs tested spans from 0 to −16 dB or −24 dB, the benefit is found to be maximal at −12 dB, after which it decreases substantially with increasing noise levels (Liu et al., 2013; Ross et al., 2007).

The identification of a special zone for audio–visual speech recognition challenges the expectations set forth by a general principle of multisensory integration known as “inverse effectiveness” (Meredith & Stein, 1986). Inverse effectiveness proposes that the benefit of concurrent multiple sensory inputs on perception should be greatest when the quality of the individual unisensory signals is poorest, that is, the greatest benefit of additional visual signals should occur at the lowest auditory SNR. Earlier reported audio–visual speech recognition studies provided support for the concept of inverse effectiveness (Erber, 1969; Sumby & Pollack, 1954). For example, Sumby and Pollack (1954) reported an inverse relationship between multisensory benefits and the quality of auditory input at auditory SNRs up to −30 dB. However, Ross et al. (2007) argued that the observed benefits at these lower SNRs were likely driven by artefacts of the task design. In Sumby and Pollack’s study, participants were exposed to checklists, prior to and during testing, which contained the spoken words. This may have allowed participants to guess the words at lower SNRs based on speech reading performance, leading to inflated audio–visual effects at these SNRs. Ross et al. (2007) alternatively argue that the sensory system is tuned to integrate multiple sensory inputs for speech primarily at intermediate SNRs (Ross et al., 2007). At the extreme ends of the noise spectrum, the system defaults to unisensory processing: relying solely on visual cues (lip-reading) when the auditory signal is highly degraded (at the lowest SNRs) or solely on auditory cues when the auditory signal is very clear (at the highest SNRs). In parallel to the special zone for audio–visual input, it is possible that the face-benefit for speech recognition in auditory-only conditions may also be most prominent at these selective SNRs, such as at −8 dB in our study. At these specific zones, engagement of the visual system, along with the corresponding behavioural face-benefit, could be maximally stimulated. This may also explain why previously observed face-benefits for speech in more optimal listening conditions have been relatively modest, ranging from gains of 0.43% (non-significant) to 2.08% (Riedel et al., 2015; Schelinski et al., 2014; von Kriegstein et al., 2008). Future work should examine face-benefits in auditory-only speech recognition at SNRs above −8 dB to ascertain whether the contribution of visual mechanisms diminishes under more noise-intensive conditions.

### Differences in the visual mechanisms underpinning the face-benefit for speech and voice-identity recognition

Paralleling effects of voice–face learning on speech recognition, the advantage gained in recognising voice-identity following voice–face learning was also evident in the noisiest listening condition, namely SNR −8 dB. Previous studies have demonstrated that manipulations of concurrent visual facial input can affect voice-identity recognition (O’Mahony & Newell, 2012; Robertson & Schweinberger, 2010; Schweinberger & Robertson, 2017; Schweinberger et al., 2007). However, to the best of our knowledge, no study has yet explored how concurrent visual input affects voice-identity recognition in noisy listening conditions, making it difficult to make direct comparisons regarding the influence of visual information on both speech and voice-identity processing.

Notably, our results showed that unlike speech processing, the benefit on voice-identity recognition for speakers previously learned by face, compared to speakers learned in the control condition, was not restricted to SNR −8 dB. Rather face-learned speakers were generally more accurately recognised. For the face-benefit participant group, this was evident in all noise levels, including those with a relatively high SNR. Moreover, in this group, there was evidence that the face-benefit (normalised score) scaled with increasing auditory noise. Why might the face-benefit in noise differ between speech and voice-identity recognition? Potentially, there may be differences in voice-identity and speech processing that reflect such findings. Specifically that our ability to recognise voice-identity is less robust than our ability to recognise others by face (Ellis et al., 1997; Hanley & Damjanovic, 2009; Hanley et al., 1998; Richard Hanley & Turner, 2000; see Stevenage & Neil, 2014 for review). It is therefore likely that interactions between the voice and face systems fundamentally support familiar voice-identity recognition (Jones & Tranel, 2001; Maguinness & von Kriegstein, 2017; von Kriegstein et al., 2006), and that such cross-modal effects are more readily observed for voice-identity recognition, even at lower SNRs, when the auditory signal is relatively salient (Schall et al., 2013; Schelinski et al., 2014; Sheffert & Olson, 2004; von Kriegstein et al., 2008).

Indeed, the face-benefit for voice-identity recognition is a robust phenomenon that has been observed under more optimal listening conditions in several studies. These investigations have employed a variety of audio–visual control learning conditions for validation, including familiarising the listener with the speaker by voice alone (Sheffert & Olson, 2004; Zäske et al., 2015) or in conjunction with other visual input including the speaker’s name (von Kriegstein & Giraud, 2006) or, as in the current study, a visual image depicting the speaker’s occupation (Maguinness & von Kriegstein, 2021; Schall et al., 2013; Schelinski et al., 2014; von Kriegstein et al., 2008). This robust benefit of voice–face learning is likely underpinned by the perceptual system’s sensitivity to the common-cause *static* identity cues available in both sensory streams. Voices are caused by physical visual structures (i.e., the vocal tract) and static voice properties, including vocal tract resonance (i.e., timbre) and fundamental frequency (i.e., pitch) which provide information about the visual structural characteristics of the speaker, including face-identity (Ghazanfar et al., 2007; Ives et al., 2005; Kim et al., 2019; Krauss et al., 2002; Mavica & Barenholtz, 2013; Oh et al., 2019; Smith & Patterson, 2005; Smith et al., 2005; Smith et al., 2016a, 2016b). These causal cross-modal relationships are rapidly acquired (Shams & Seitz, 2008; von Kriegstein et al., 2008), facilitating subsequent auditory-only recognition processing at a speaker-specific level (Blank et al., 2015). In addition, dynamic (i.e., change over time or spatiotemporal) cues in the face and voice also share common source identity information (Kamachi et al., 2003; Lachs & Pisoni, 2004; Mc Dougall, 2006; Simmons et al., 2021; Smith et al., 2016b). Recently, we demonstrated that these “dynamic facial signatures” (O’Toole et al., 2002; Roark et al., 2003), learned during audio–visual exposure, may be additionally recruited during auditory-only voice-identity recognition in challenging listening conditions (Maguinness & von Kriegstein, 2021). This complementary dynamic mechanism may be particularly engaged when static identity cues in the sensory signal are degraded, providing a “backup” support, rather than fundamental, system for voice-identity recognition (Maguinness & von Kriegstein, 2021).

In contrast, the face-benefit for speech recognition has been less investigated and, when observed, tends to be smaller than the face-benefit for voice-identity recognition (Riedel et al., 2015; Schelinski et al., 2014; von Kriegstein et al., 2008), as in this study where benefits of less than 1% were observed in all noise levels, except for SNR −8 dB. Although it is possible that the engagement of the visual system may be less apparent for speech recognition at lower SNRs, it is also important to consider that these relatively small face-benefits (< 1%) may also be expected given the high recognition rates in these lower noise levels, where recognition performance was markedly stable and robust (> 97% correct, Table 2). This robust near ceiling performance may have been influenced by the SNR ranges tested, which may potentially not have been challenging enough to impact speech recognition. Indeed, only in SNR −8 dB did noise significantly impact speech recognition performance; the speech utterances of voice–occupation-learned speakers were recognised more poorly than that of speakers learned by face, that is, a face-benefit was apparent. Employing a lower SNR (−10 dB), however, led to chance performance in pilot experiments in which participants were exposed to a single noise level during auditory-only testing. Another possibility is that recognition performance may have been inflated due to sentence repetition. All 30 sentences were heard in each of the different noise levels. It is possible that, if a sentence is first heard in a high SNR and is then repeated in a lower SNR, it may still be understandable at the lower SNR. Top-down factors such as general priming effects or familiarity with specific sentences may make detecting task effects of interest (e.g., learning condition) more difficult. In addition, the audio–visual training paradigm used in Experiment 2 was focused on recognising the identity of the speaker. Training that emphasises the processing of the dynamic speech signal may also impact subsequent speech recognition performance for face-learned speakers (Lidestam et al., 2014; Rosenblum et al., 2007).

### Inter-individual variability in the face-benefit

Although the face-benefit appears to be a supportive mechanism for auditory-only processing, it is important to note that not all individuals who participated in the current study displayed a face-benefit. The increase in the face-benefit with noise, for both auditory-only speech and voice-identity recognition, was restricted to those who benefitted overall (i.e., average positive benefit across noise levels) from voice–face learning. The number of participants who showed this overall face-benefit in the auditory-only speech and voice-identity recognition tasks is on par with previous studies (e.g., von Kriegstein et al., 2008). Currently, it is unclear why some individuals do not benefit from multisensory learning or exposure to multisensory, rather than unisensory, stimuli (see Mathias & von Kriegstein, 2023; Matusz et al., 2017; Murray & Shams, 2023 for reviews on multisensory learning, including individual differences). In general, individual differences in learning outcomes have been observed in a range of multisensory tasks in which concurrent sensory cues are available (de Haas et al., 2012; Gurler et al., 2015; Hirst et al., 2020; Mallick et al., 2015). Previous studies suggest that there is no correlation between the face-benefits for speech and voice-identity recognition in neurotypical participants (von Kriegstein et al., 2008). For example, a participant with a high face-benefit for speech will not necessarily also exhibit a high face-benefit for voice-identity recognition, indicating that the face-benefit is unlikely to be governed by a domain general mechanism. Moreover, this suggests that post-learning tasks used to assess learning benefits might produce varying estimates of multisensory advantages. Findings from autism spectrum disorder suggest that the face-benefit for auditory-only speech processing may relate to differences in lip-reading abilities (Maguinness & von Kriegstein, 2017; von Kriegstein et al., 2006, 2008). In addition, findings from developmental prosopagnosia (McConachie, 1976), a severe deficit in face-identity processing, implicate intact face-identity processing as a necessary component for the face-benefit on voice-identity processing (Maguinness & von Kriegstein, 2017; von Kriegstein et al., 2006, 2008). Such findings imply that individual differences in specific visual abilities may potentially relate to the differences in face-benefits observed between participants in the current study.

Notably, in line with previous observations (von Kriegstein et al., 2008), we noted that the face-benefit was less prevalent in our participant samples for auditory-only speech, than voice-identity, recognition. Although we propose that cross-modal interactions may be fundamental to supporting voice-identity recognition, potentially for speech recognition, the speaker-specific benefit of visual information may be less apparent owing in part to the nature of the task. Speech processing involves recognising as common a speech sound (e.g., /a/, /i/, etc.) spoken by different speakers (von Kriegstein et al., 2010). As such, between-speaker variability in speech (visual and auditory cues) may be less apparent than that observed for voice-identity recognition—a task which fundamentally requires detecting *differences* between speakers in static identity cues. We propose, that at the individual level, the ability to perceive the visual lip movements of a specific speaker likely translates into how precise a generative model for this specific speaker will be. We speculate that participants with a less precise generative model about the speaker-specific articulatory dynamics will profit less from this predictive information, in the context of auditory-only speech processing. In the current study, however, we did not collect direct measures of lip-reading or face-identity recognition performance. Furthermore, different groups of participants completed the two experiments. Further studies examining these measures across sensory modalities may assist in determining how inter-individual variability may arise in the face-benefit, if, or how, it may vary as a function of auditory noise, and other factors, such as learning duration, task objectives, and the nature of learning—be it implicit or explicit. Additional behavioural measures, such as eye-tracking during audio–visual learning, may also be revealing. Such measures have been useful in examining, for instance, which aspects of the face are important to attend to for identity processing and how this can vary across different participant samples—from developmental prosopagnosia to super recognisers (Bobak et al., 2017).

### The face-benefit: facilitating recognition beyond auditory noise

Challenging listening conditions surround us daily, such as when we speak with someone on the phone in the presence of background noise, or in the context of multiple speakers. Listening conditions are not only challenging due to extrinsic factors; auditory processing can also be impacted due to developmental disorders (e.g., developmental dyslexia or auditory processing disorder) or hearing loss, including that which accompanies advancing age (Iliadou et al., 2017; Kral & Sharma, 2023; Pichora-Fuller & Souza, 2003; Roth et al., 2011; Ziegler et al., 2009). Indeed, among older adults, one of the most reported perceptual difficulties is the processing of speech, particularly in challenging listening environments (Pichora-Fuller, 2008; Schneider et al., 2002; Sheldon et al., 2008; Sommers, 1997; Surprenant, 2007). In alignment with findings on concurrent visual input (Maguinness et al., 2011; Sommers et al., 2005), our results suggest that auditory processing in such populations could benefit from audio–visual voice–face learning, aiding in the resolution of noisy auditory input. Several studies have demonstrated that older (Holmes et al., 2018), and also younger (Kreitewolf et al., 2017), adults are more accurate at recognising speech utterances from familiar talkers (Johnsrude et al., 2013) and that this benefit is greatest in adverse listening conditions (Souza et al., 2013). It is possible that this gain may be mediated by the recruitment of additional visual mechanisms (in cases where the familiar speakers were also known by face), which may assist in enhancing the predictability of the auditory signal.

## Conclusion

In summary, we replicated the face-benefit for auditory-only speech and voice-identity recognition in a similar proportion of participants as in previous studies. The face-benefit for voice-identity recognition was significant also over the whole group, while the face-benefit for speech was not. Importantly, we showed, for the first time, that the visual information acquired during audio–visual learning is used in an adaptable manner, tailored to task demands, to enhance subsequent auditory-only recognition in challenging listening conditions. These findings corroborate and advance an audio–visual model of human auditory communication. They suggest that the brain can develop a remarkably flexible mechanism for enhancing auditory processing considering sensory demands and again point towards inter-individual differences in the utilisation of learned visual information.

## Supplemental Material

sj-docx-1-qjp-10.1177_17470218241278649 – Supplemental material for Prior multisensory learning can facilitate auditory-only voice-identity and speech recognition in noise Supplemental material, sj-docx-1-qjp-10.1177_17470218241278649 for Prior multisensory learning can facilitate auditory-only voice-identity and speech recognition in noise by Corrina Maguinness, Sonja Schall, Brian Mathias, Martin Schoemann and Katharina von Kriegstein in Quarterly Journal of Experimental Psychology
