# Supplementary material for: Prior multisensory learning can facilitate auditory-only voice-identity and speech recognition in noise
Source: Q J Exp Psychol (Hove). 2024 Sep 20;78(7):1348–68. doi: 10.1177/17470218241278649 (PMC12181647; doi:10.1177/17470218241278649)
Supplement: sj-docx-1-qjp-10.1177_17470218241278649 – Supplemental material for Prior multisensory learning can facilitate auditory-only voice-identity and speech recognition in noise [file sj-docx-1-qjp-10.1177_17470218241278649.docx]

Supplementary Material for:

Prior multisensory learning can facilitate auditory-only voice-identity and speech recognition in noise

Corrina Maguinness*^1,2^, Sonja Schall^2^, Brian Mathias^1,3^, Martin Schoemann^4^, & Katharina von Kriegstein^1,2^

*1 Chair of Cognitive and Clinical Neuroscience, Technische Universität Dresden, Dresden, Germany*

*2 Max Planck Institute for Human Cognitive and Brain Sciences, Leipzig, Germany*

*3 School of Psychology, University of Aberdeen, Aberdeen, United Kingdom*

*4 Chair of Psychological Methods and Cognitive Modelling, Technische Universität Dresden, Dresden, Germany*

*****corrina.maguinness@tu-dresden.de; sonjaschall@gmail.com; brian.mathias@abdn.ac.uk; martin.schoemann@tu-dresden.de; katharina.von_kriegstein@tu-dresden.de

*****Corresponding author

Supplementary Analyses

Experiment 1

Data Analysis

For the face-benefit participant group (*N* = 14) we also examined response time data using the same procedure applied to the accuracy data. That is, we used linear mixed effects (LME) regression analyses. The fixed effects of learning (voice-face learned, voice-occupation learned) and noise level (SNR +4, 0, -4, -8 dB) were coded and treated as per the accuracy analysis. The random effects structure was again selected using backward model selection. The final model included a random intercept by participant; note this is an identical structure to the accuracy LME analysis. This additional analysis served to determine if auditory-only speech recognition was facilitated in terms of both accuracy and response time for voices learned together with faces compared to the audio-visual control condition, that is, that the face-benefit could not be explained by a speed-accuracy trade-off.

Results

Response time: auditory-only speech recognition

*Face-benefit participant group*

The linear mixed effects model on response times for the face-benefit participants (N = 14) revealed an effect of noise level (*B* = -2.32, *SE* = 0.51, *t* = -4.54, *p* < 0.001, 95% CI [-3.33 – -1.30]). Post hoc analyses revealed response times were slowest in SNR -8 dB (*M*_RT_ = 703 ms; SD = 22 ms) compared to all other noise levels (all *p*s < 0.01). There was no significant effect of learning (*p* = 0.26) or interaction between the effects of learning and noise level (*p* = 0.28). Rather the variance in response times was largely captured by the random effect of participant. The full model results, including *R*^2^ values, are shown in Table S1. As such, the face-benefit observed for auditory-only speech recognition accuracy in this group (see main manuscript for analysis) could not be explained by faster responses to voice-occupation, than voice-face, learned speakers i.e., no speed-accuracy trade-off was present. See Table S2, left of Table, for the mean response times for each of the learning conditions and noise levels for auditory-only speech recognition.

**Table S1.** Summary of the linear mixed effects analysis testing the effect of learning condition and noise level on auditory-only speech recognition response times in Experiment 1 for participants with a positive overall face-benefit (*N* = 14).

| **Auditory-only Speech Recognition** | | | | | | |
| --- | --- | --- | --- | --- | --- | --- |
| **Linear Mixed Effects Regression: Response Times (Face-benefit Participants)** | | | | | | |
| Fixed effects | *B* | 95% CI | *SE* | *t* | *p* | |
| Intercept | 679.53 | 620.60 – 738.47 | 29.73 | 22.86 | | <0.001 |
| Learning | -5.68 | -15.58 – 4.23 | 4.99 | -1.14 | | 0.26 |
| Noise level | -2.32 | -3.33 – -1.30 | 0.51 | -4.54 | | <0.001 |
| Learning x Noise level | 1.10 | -0.92 – 3.12 | 1.02 | 1.08 | | 0.28 |
| Random effects |  | Variance | *SD* |  | |  |
| Participant | Intercept | 12284.48 | 110.84 |  | |  |
| Marginal *R*^2^ = 0.01; Conditional *R*^2^ = 0.96  *N* = 14 | | | | | | |

**Table S2**. **Response time performance for face-benefit participants for auditory-only speech recognition (Experiment 1; *N* = 14) and auditory-only voice-identity recognition (Experiment 2; *N* = 19).** Mean response times (in milliseconds with standard deviations) for voice-face and voice-occupation learned speakers, for each of the four noise levels.

|  |  |  |  |  |  |  |  |  |
| --- | --- | --- | --- | --- | --- | --- | --- | --- |
|  | **Auditory-only speech recognition** | | | | **Auditory-only voice-identity recognition** | | | |
|  | **Face-benefit participants (*N*=14)** | | | | **Face-benefit participants (*N*=19)** | | | |
| **signal-to-noise ratio** | **+4 dB** | **0 dB** | **-4 dB** | **-8 dB** | **+4 dB** | **0 dB** | **-4 dB** | **-8 dB** |
|  | | | | | | | | |
| **voice-face** | 676  (129) | 673  (115) | 671  (109) | 701  (129) | 710  (156) | 691  (167) | 708  (145) | 729  (152) |
| **voice-occupation** | 670  (112) | 685  (115) | 693  (118) | 705  (112) | 754  (167) | 753  (146) | 771  (173) | 810  (158) |

Experiment 2

Data Analysis

For the face-benefit participant group (*N* = 19), we examined response times using the same procedure as per Experiment 1, with the exception that response times for auditory-only voice-identity recognition were examined. Fixed effects were again learning (voice-face learned, voice-occupation learned) and noise level (SNR +4, 0, -4, -8 dB), coded and treated as per the accuracy analysis (see main manuscript). Random effects were again selected using backward model selection. The final model included a random intercept of participant and a participant-by-learning condition slope; note this is an identical structure to the accuracy LME model.

Response time: auditory-only voice-identity recognition

*Face-benefit participant group*

The linear mixed effects model on response times for the face-benefit participants (*N* = 19) revealed an effect of learning (*B* = -56.90, *SE* = 13.82, *t* = -4.12, *p* < 0.001, 95% CI [-84.22 – -29.58]). Voice-identity recognition was faster for speakers previously learned in the voice-face condition (*M*_RT_ = 709 ms; *SD* = 18 ms), compared to speakers learned in the voice-occupation condition (*M*_RT_ = 772 ms; SD = 18 ms). An effect of noise level was also evident (*B* = -3.23, *SE* = 0.97, *t* = -3.34, *p* = 0.001, 95% CI [-5.14 – -1.32]). Response times for the highest noise level i.e., SNR -8 dB (*M*_RT_ = 769 ms; SD = 26 ms) were slower than SNR 0 dB (*p* = 0.001) (*M*_RT_ = 722 ms; *SD* = 26 ms) and SNR +4 dB (*p* = 0.02) (*M*_RT_ = 732 ms; *SD* = 26 ms). There was no interaction between learning and noise level (*p* = 0.16). As per the accuracy LME model on this face-benefit group (see main manuscript), the marginal and conditional *R*^2^ values indicated that variance explained by individual differences between participants in terms of the effect of learning on response times for voice-identity recognition substantially exceeded the variance explained by the model’s fixed effects. The full model results are shown in Table S3. Taken together, the results point towards inter-individual variability, however, they demonstrate that the face-benefit cannot be explained by a speed-accuracy-trade off in performance. Rather they suggest that prior audio-visual voice-face (in comparison to voice-occupation) learning benefits subsequent auditory-only voice-identity recognition both at the level of accuracy *and* response time. See Table S2, right of Table, for the mean response times for each of the learning conditions and noise levels.

**Table S3.** Summary of the linear mixed effects analysis testing the effect of learning condition and noise level on auditory-only voice-identity recognition response times in Experiment 2 in participants with a positive overall face-benefit (*N* = 19).

| **Auditory-only Voice-identity Recognition** | | | | | | |
| --- | --- | --- | --- | --- | --- | --- |
| **Linear Mixed Effects Regression: Response Times (Face-benefit Participants)** | | | | | | |
| Fixed effects | *B* | 95% CI | *SE* | *t* | *p* | |
| Intercept | 734.17 | 668.48 – 799.87 | 33.24 | 22.09 | | <0.001 |
| Learning | -56.90 | -84.22 – -29.58 | 13.82 | -4.12 | | <0.001 |
| Noise level | -3.23 | -5.14 – -1.32 | 0.97 | -3.34 | | 0.001 |
| Learning x Noise level | 2.76 | -1.06 – 6.58 | 1.93 | 1.43 | | 0.16 |
| Random effects |  | Variance | *SD* |  | |  |
| Participant | Intercept | 20563.66 | 143.40 |  | |  |
|  | Learning | 1929.56 | 43.93 |  | |  |
| Marginal *R*^2^ = 0.05; Conditional *R*^2^ = 0.89  *N* = 19 | | | | | | |
